# Fatal acute hepatic failure in a family infected with the hepatitis A virus subgenotype IB: A case report

**DOI:** 10.1097/MD.0000000000007847

**Published:** 2017-09-01

**Authors:** Yuichi Yoshida, Yohei Okada, Akiko Suzuki, Keisuke Kakisaka, Yasuhiro Miyamoto, Akio Miyasaka, Yasuhiro Takikawa, Tsutomu Nishizawa, Hiroaki Okamoto

**Affiliations:** a Division of Hepatology, Department of Internal Medicine, Iwate Medical University School of Medicine, Morioka, Iwate; b Division of Virology, Department of Infection and Immunity, Jichi Medical University School of Medicine, Shimozuke, Tochigi, Japan.

**Keywords:** acute liver failure, entire genomic sequence, fulminant hepatitis, hepatitis A virus, subgenotype IB

## Abstract

**Rationale:**

Hepatitis A viral infection is a well-known cause of subclinical or acute self-limited hepatitis. Few cases of hepatitis A virus (HAV)–associated acute liver failure (ALF) have been reported in low HAV endemic countries annually.

**Patients concerns:**

To investigate the possible factors that affected the severity of HAV infection, a family cluster infected with the HAV subgenotype IB strain, which is not common in Japan, was described.

**Diagnoses:**

This family consisted of five members who all were infected with HAV.

**Interventions:**

Four of the five patients hospitalized except for an asymptomatic patient.

**Outcomes:**

Two of the five patients, men in their 50s and 60s, developed ALF, and one patient died. Various host factors, including sex (male), age, and a high bilirubin level, may affect the outcomes. Based on viral factors, HAV RNA was higher in the fatal case compared with others, and it decreased within a short period of time. The similarity of the nucleotide sequences was 99.9% among the HAV isolates based on an entire genomic sequence. Deletions and/or insertions on the HAV protein-coding sequences that caused a frameshift were found in surviving cases but not in the fatal case.

**Lessons:**

The rapid clearance of increased HAV and the absence of defective HAV might be closely associated with the onset of liver failure.

## 1 Introduction

Hepatitis A virus (HAV) is a single-stranded RNA virus, and it belongs to the *Picornaviridae* family. HAV strains isolated from different parts of the world show a modest degree of genetic diversity, and they have been classified into 6 genotypes (I–VI), of which genotypes I, II, and III are found in humans. These are then further divided into subgenotypes IA and IB, IIA and IIB, and IIIA and IIIB, respectively.^[1]^ HAV is a major cause of acute hepatitis worldwide and it is transmitted via the fecal–oral route. Sporadic infection and outbreaks of hepatitis A are not common in developing and developed countries. Over 100 patients who were infected with the same HAV strain have often been reported in endemic countries.^[2]^ On the contrary, although 342 Japanese patients were infected during an outbreak of infection with HAV subgenotype IA, which is a major strain in Japan, in 2014,^[3]^ only a few cases of HAV-associated acute liver failure (ALF) were reported during the same year in the Japan nationwide survey of ALF and late-onset hepatic failure. In the United states, the mortality rate of hepatitis A in the general population is 0.2%, and a 1% increase is observed in patients who are over 49 years of age.^[4,5]^ Therefore, hepatitis A is a well-known cause of subclinical or acute self-limited hepatitis in the general population and severe hepatitis, including HAV-associated ALF, which is rare.

Herein, we report a family that acquired a cluster infection with the HAV subgenotype IB strain, which is not common in Japan. This family showed a high rate of ALF and mortality as 2 of the 5 members developed ALF, and 1 patient died.

## 2 Case report

Herein, we describe a family of 3 men and 2 women who lived in Akita prefecture, Tohoku district of northern Japan. The clinical manifestations and prognoses of this family are shown in Table 1. In January 2015, a 60-year-old woman (patient 1) presented with jaundice and general fatigue. She was admitted to a local hospital and diagnosed with hepatitis A. She had a prothrombin time (PT) of 83.9% and received steroid pulse therapy due to acute hepatitis, and she successfully recovered. Ten days after patient 1 presented with symptoms, a 34-year-old man (patient 2), the son of patient 1, presented with symptoms, such as vomiting and anorexia, and a prolonged PT of 45.9% was obtained. Patient 2 was admitted to the same hospital, and he was successfully treated with steroid pulse therapy because of severe acute hepatitis. A 63-year-old man (patient 3), who is the husband of patient 1, developed jaundice, and a PT of 11.3% was obtained. Patient 3 was admitted to the same hospital 15 days after patient 2 showed signs and symptoms of infection and treated with steroid pulse therapy due to ALF. He also successfully recovered. In February 2015, 17 days after patient 3 was admitted, a 59-year-old man (patient 4), who is the brother of patient 3, visited an outpatient clinic and complained of general fatigue, anorexia, diarrhea, and brown-colored urine. He had elevated liver enzyme levels and renal dysfunction, and laboratory results showed the following: aspartate aminotransferase 7096 U/L; alanine aminotransferase 8030 U/L; total bilirubin 7.1 mg/mL; PT 12.1%; and creatinine 3.22 mg/dL. Moreover, he was negative for hepatitis B virus surface antigen, anti-hepatitis C virus antibody, and anti-hepatitis E virus IgA antibody. Because patients 1, 2, and 3, who all also lived with patient 4, were positive for anti-HAV IgM antibodies, he was therefore suspected to have HAV-associated ALF. The risk score calculated via the early prediction model of the short-term development of hepatic encephalopathy in patients with acute liver disease was 70.9%.^[6]^ He was immediately transferred and admitted to Iwate Medical University Hospital, the center for ALF treatment in the northern part of Honshu, main island of Japan. He received steroid pulse therapy and was treated with recombinant thrombomodulin, antithrombin III and albumin preparations, platelet concentrates, and fresh frozen plasma, because of complications such as hepatorenal dysfunction and disseminated intravascular coagulation (DIC). A day after admission, continuous hemodiafiltration (CHDF) treatment was started because of the development of grade 3 hepatic encephalopathy. During CHDF treatment, his blood pressure decreased and anemia rapidly progressed, and enhanced computed tomography revealed intra-abdominal hematoma caused by spontaneous iliac arterial bleeding. The bleeding subsided after performing transarterial embolization. Hepatic encephalopathy progressed to grade 4 two days after admission, and uncontrollable arterial bleeding was observed in another region of the abdomen due to liver failure and DIC. As a result, patient 4 died 3 days after admission.

**Table 1:**
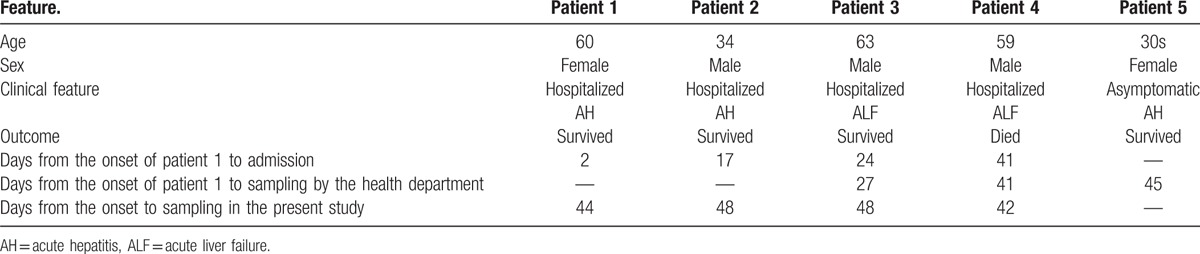
Clinical manifestations of the present family.

**Figure 1:**
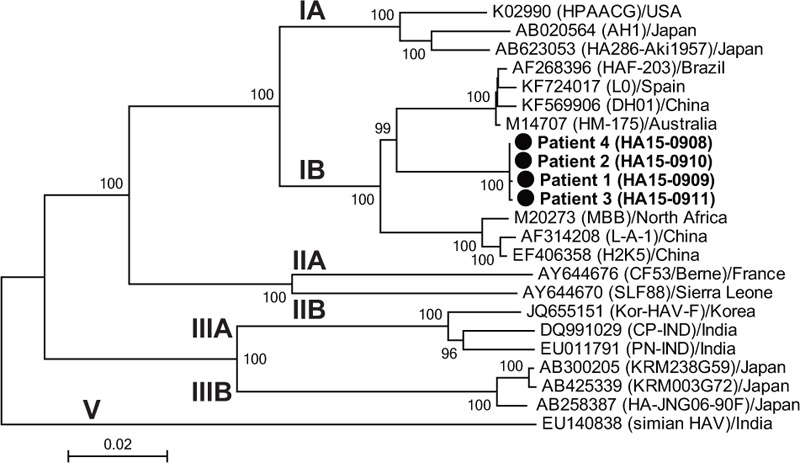
A neighbor-joining tree of the entire or near-entire genomic sequence of the four hepatitis A virus (HAV) isolates obtained in the present study with 18 reported HAV sequences of subgenotypes IA, IB, IIA, IIB, IIIA, and IIIB whose entire sequences are known and the simian HAV isolate of genotype V set as an out-group. Each reference sequence is shown with the accession number, followed by the isolate name in parenthesis and the name of the country from where it was isolated. The subgenotype IB HAV isolates obtained in the present study are shown in bold typeface and marked with a closed box. The bootstrap values (>70%) are indicated for the nodes as a percentage of the data obtained from 1000 resamplings. The scale bar is in units of nucleotide substitutions per site.

Anti-HAV IgM antibody was found in the sera obtained not only from patients 1 to 4, but also from the daughter of patient 1 who had no symptoms (patient 5). All 5 patients ate raw shellfishes 11 days before patient 1 presented with symptoms of the infection. Because the leftover of the shellfish was discarded, the source of HAV infection was not identified. Because more than 1 month had passed from the day that the shellfish in question had been eaten until the onset of the disease in patient 4 (see Table 1) was observed, a secondary infection among the family members was suspected. According to the notification of familial HAV infection, the present family was investigated by the health department of Akita prefecture, and viral loads of HAV in the sera from patients 3, 4, and 5, which were obtained shortly after the onset of symptoms, were measured via quantitative polymerase chain reaction (PCR). Patients 3, 4, and 5 were infected with the HAV subgenotype IB, and serum HAV RNA values of 4.8, 6.6, and 3.7 log copies/mL, respectively, were obtained. We obtained serum samples from all patients except for patient 5 within 10 to 44 days from the onset of symptoms, and these sera were frozen at −80°C. HAV RNA was detected in all serum samples of patients 1, 2, 3, and 4. The titers of HAV RNA from patients 1, 2, 3, and 4 were 1.1, 1.3, 0.9, and 2.0 log copies/mL, respectively. The entire genomic sequence was determined for the HAV isolates obtained from patients 1 to 4 according to the previously described method.^[7]^ The HAV subgenotype IB was found in all sera, including those from patients 1 and 2. The 4 HAV isolates shared 99.9% of the nucleotide (nt) sequence identities, but were only 94.6% to 95.7% similar to the reported subgenotype IB isolates over the entire genome. A phylogenetic tree constructed based on the entire genome revealed that the 4 HAV isolates obtained in the present study were segregated into a cluster within subgenotype IB (Fig. 1). Complete genes encoding whole HAV proteins were detected in the HAV isolate obtained from patient 4 (HA15–0908) (Table 2). However, defective HAV genes, which had deletion(s) and/or insertion(s) on the HAV protein coding sequences that caused a frameshift, were detected. Patient 1 had the HAV with an insertion of 1 nt and 3 deletions of 1, 1, and 813 nts. Patients 2 and 3 had the HAVs with 1 and 3 mixed nts of wild-type and a deletion of nt. This study was approved by Iwate Medical University Ethics Committee (H26-139). Informed consent was also obtained.

**Table 2:**
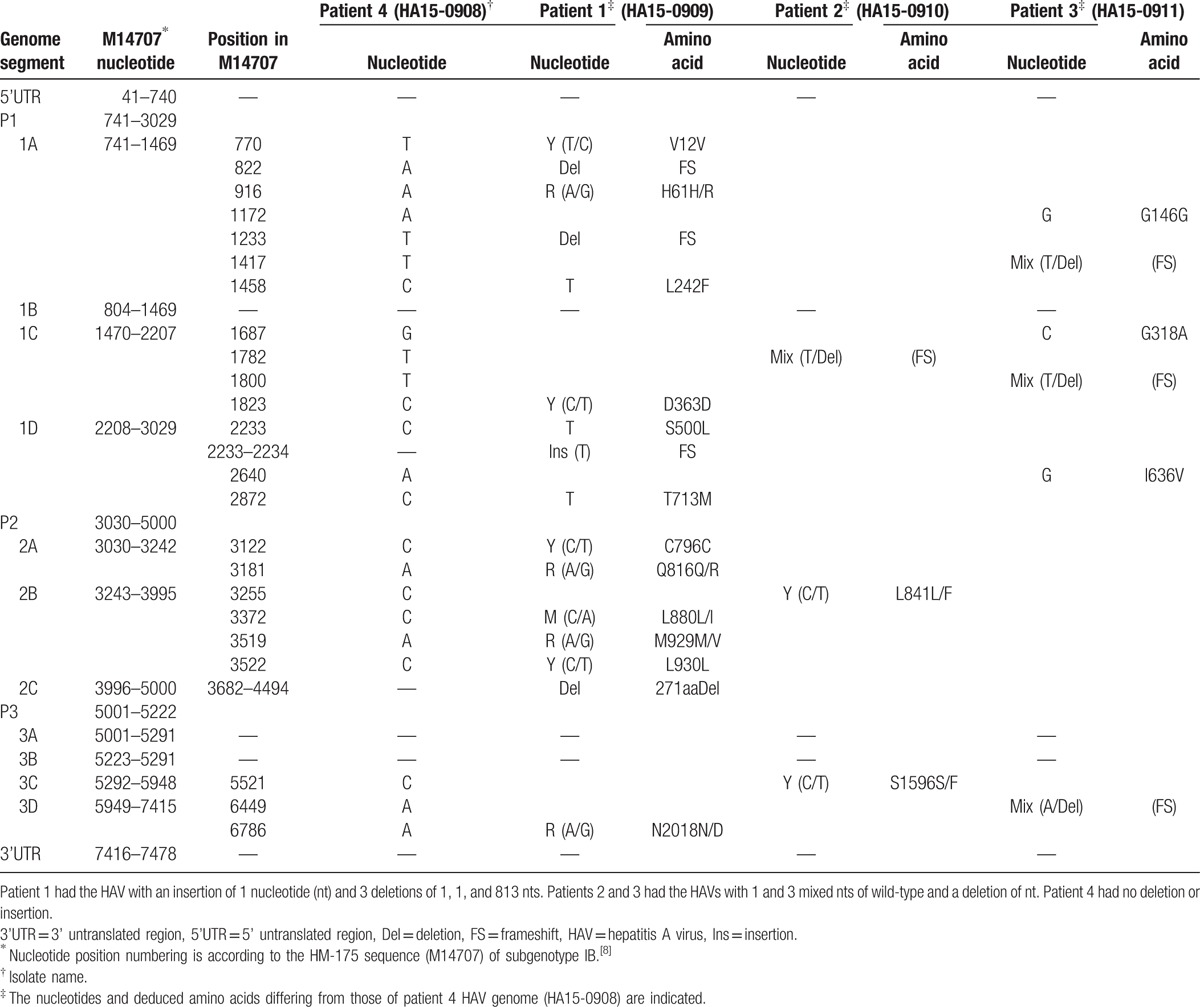
Deletions and insertions on the HAV protein coding sequences detected in the sera of the present family.

## 3 Discussion

All 5 members of the family were infected with the HAV subgenotype IB strain, which has caused a sporadic hepatitis A in Japan. A woman in her 60s (patient 1) and a man in his 30s (patient 2) were treated before the onset of ALF, and they survived. The condition of the patients in their 50s (patient 4) and 60s (patient 3) progressed to HAV-associated ALF, and the former died. Only 1 patient (patient 5) had subclinical hepatitis, whereas all others had been hospitalized. Although most adult patients infected with HAV develop self-limited acute hepatitis, HAV-associated ALF has annually been reported in Japan. HAV-related mortality rates of 0.02 per 100,000 population per year in the United States^[9]^ and 16.8 per 1000 hospitalizations according to a Taiwan nationwide cohort study were reported.^[10]^ According to a nationwide survey in Japan, 14 patients developed HAV-associated fulminant hepatitis, thus indicating ALF with hepatic encephalopathy, and 6 patients (42.8%) either died or underwent liver transplant during the 5-year study period.^[11]^ In the present study, the severity of hepatic injury was greater than that reported in a previous study.^[1]^ We investigated the possible factors that affected the severity of HAV infection in the present family.

Host factors, including sex (male), age (50 years or older), a high bilirubin level, underlying chronic liver disease, and the length of stay in the hospital, may increase the likelihood of a fulminant course or death.^[10,12]^ Two male patients in their 50s and 60s had ALF complication, and hyperbilirubinemia and renal dysfunction were observed in the fatal case. The accumulation of host factors correlated with the severity of hepatitis A. However, this hypothesis does not fully explain the extremely high rate of HAV-associated ALF in the present study. Not only the already known host factors, but also some viral factors and/or indeterminate host factors, which may be associated with the host immune response or hepatic regeneration, might be involved in the onset of HAV-associated ALF in the present family.

Some viral factors, such as HAV load, a higher rate of substitution in the 5’ untranslated region (UTR), and P2 region encoding nonstructural protein 2B and 2C of the HAV genome, are associated with an increased frequency of HAV-associated ALF.^[13]^ Rezende et al^[12]^ reported that a marked reduction in the HAV load was detected in the sera of HAV-associated ALF patients. However, a study conducted by Fujiwara et al^[14]^ revealed that HAV RNA was detected quantitatively in the majority of the sera of hepatitis A cases, not only in the acute phase but also in the early convalescent phase. In addition, a higher viral replication was found in severely infected patients via real time RT-PCR. Thus, a higher viral replication at onset may induce an excessive host response and severe diseases due to the rapid reduction of the HAV load.^[15]^ In the present family study, the HAV load of patient 4 was higher than that in patients 3 and 5, and it decreased within a short period of time.

The association between the severity of hepatitis A and HAV subgenotypes has not been established. Rezende et al^[12]^ presented that fulminant hepatitis is associated with the HAV genotypes, except for IA in 50 cases infected with HAV. Ajmera et al^[16]^ deemed that a poor outcome in HAV-associated ALF is related to a rapid viral clearance. However, age, sex, nt substitutions in 5’ UTR, and HAV subgenotype based on the study of 29 HAV-associated ALF patients were not associated with the condition. Interestingly, subgenotype IB was found more frequently among the ALF cases (16.7%, 4 cases of IB/24 cases whose HAV subgenotype were determined) compared with the nonliver failure cases in the United States (2.2%, 19 cases of IB/834 cases of hepatitis A).^[16]^ Although genetic variations of HAV including genotypes and nt substitutions in 5’ UTR are associated with ALF, the relationship between the occurrence of the defective HAV protein-coding region and the outcome should be investigated. Patients 1, 2, and 3 had some nt substitutions in the regions encoding structural and nonstructural proteins (patients 1, 2, and 3) compared with the fatal ALF patient (patient 4) with a complete HAV sequence (see Table 2). In the sera of patient 1, a long deletion of 813 nt was seen in the HAV nonstructural protein 2B and 2C sequences. The 2B and 2C sequences are important for cell culture adaptation and HAV replication.^[17,18]^ From the time of onset, the frequency of insertion or deletion increased. HAV in patient 1 had 1 insertion and 3 deletions, HAVs in patients 2 and 3 had 1 or 3 deletions, and HAV in patient 4 did not have any deletions. It was estimated that patient 1 had been infected with HAV that has a complete genome because this patient was a suspected source of the secondary infection among the family. Considering that the defective HAV sequence of patient 1 was detected in the convalescent phase, insertion and deletion occurred from the acute to the early convalescent phase. The accumulation of these HAV genomic mutations associated with viral replication and protein expression might weaken the immune reaction, and whether or not such mutations on the HAV protein-coding region played a role in the disease onset should be further studied. To explain these findings, we hypothesized the following: first, in the early phase of HAV infection, complete sequences that encode all viral proteins were amplified in the HAV-infected liver cells and HAV RNA was detected in the sera. Secondly, as the activated reaction of the host immune system reduced the amplification of complete HAV, defective and dysfunctional HAV became predominant during HAV elimination. Third, in the patient, a severe reaction between hepatocytes producing complete HAV and the host immune system occurred, and HAV was rapidly eliminated before producing a defective gene, although the replacement of complete HAV for defective HAV was observed to slowly progress in the patient without fatal ALF complication. Not only the rapid clearance of increased HAV but also the absence of defective HAV-protein coding genes might be associated with the development of ALF with hepatic encephalopathy. This concept is speculative and should be verified via a sequential study using the sera from numerous patients with hepatitis A, including those with ALF.

## 4 Conclusions

In conclusion, we identified the family infected with HAV subgenotype IB. Although age and sex may play an important role in the onset of ALF, the rapid clearance of increased HAV and the absence of any defective HAV might be associated with the onset of severe liver failure.
